# Semiparametric outcome regression-based estimator of Mann–Whitney-type causal effect

**DOI:** 10.1186/s12874-026-02840-1

**Published:** 2026-03-26

**Authors:** Safiya S. Sani, Bryan S. Blette, Chun Li, Abubakar Yahaya, Hussaini G. Dikko, Abubakar Usman, Usman J. Wudil, Faisal Dankishiya, Nafiu Hussaini, C. William Wester, Muktar H. Aliyu, Bryan E. Shepherd

**Affiliations:** 1 Department of Agronomy, Ahmadu Bello University (ABU), Zaria, Kaduna, Nigeria; 2 Department of Biostatistics, Vanderbilt University Medical Center (VUMC), Nashville, TN USA; 3 Department of Population and Public Health Sciences, University of Southern California, Los Angeles, CA USA; 4 Department of Statistics, Ahmadu Bello University (ABU), Zaria, Kaduna, Nigeria; 5 Vanderbilt Institute for Global Health (VIGH), 2525 West End Avenue, Suite 750, Nashville, TN 37203-1738 USA; 6 Department of Medicine, Aminu Kano Teaching Hospital (AKTH), Kano, Nigeria; 7 Department of Mathematical Sciences, Bayero University Kano (BUK), Kano, Nigeria; 8 Department of Medicine, Division of Infectious Diseases, Vanderbilt University Medical Center (VUMC), Nashville, TN 37203-1738 USA; 9 Department of Health Policy, Vanderbilt University Medical Center (VUMC), Nashville, TN USA

**Keywords:** Albuminuria, Causal effect estimation, Cumulative probability model, Estimated Glomerular filtration rate, Mann–Whitney-type parameter, Semiparametric models, Urine albumin to creatinine ratio

## Abstract

We introduce a novel semiparametric estimator for Mann–Whitney-type causal effects based on the cumulative probability model (CPM). CPMs are rank-based, invariant to monotone transformations of the outcome, and offer flexible outcome regression under confounding. We formalize the estimation under causal consistency, no interference, ignorability, and positivity assumptions, and develop accompanying inference procedures. Through simulations with varying sample sizes and effect magnitudes, the CPM estimator shows reduced variability and improved predictive accuracy relative to mis-specified parametric transformations. We demonstrate its applicability in a large cohort of people with HIV (PWH) in Northern Nigeria by assessing the causal effect of HIV status on albuminuria levels. Overall, our results highlight the value of robust semiparametric methods for causal inference in observational settings beyond average treatment effects. Findings should be interpreted in light of the observational design and the potential for unmeasured confounding.

**Supplementary Information:**

The online version contains supplementary material available at 10.1186/s12874-026-02840-1.

## Introduction

The Neyman–Rubin Causal Model [9, 26] defines causal effects through potential outcomes: for each subject, $\mathrm{Y(1)}$ and $\mathrm{Y(0)}$ denote the outcomes under treatment and control, respectively. Because only one of these can be observed, individual causal effects are not identifiable, and inference focuses on population-level estimands such as the Average Causal Effect (ACE) or contrasts of distributional summaries [13]. While mean-based estimands dominate causal inference, there is increasing interest in effect measures that capture broader distributional effects. The Mann–Whitney (MW) or Wilcoxon rank-sum test compares entire outcome distributions and yields the probabilistic index—the probability that a randomly selected treated subject has a higher outcome than a randomly selected control. This parameter is robust to skewness, invariant to monotone transformations, interpretable across diverse outcome types, and valid with ties [4]. Extensions to permit covariate adjustment with probabilistic index models [31] incorporate auxiliary information but do not provide a formal causal framework for analysis.

Recent work by Fay et al. [5] and Zhang et al. [38] introduced a Mann–Whitney-type causal estimand, $\theta \mathrm{=}P\left({Y}_{i}\left({1}\right)\mathrm{>}{Y}_{j}\left({0}\right)\right)+\frac{1}{2}P\left({Y}_{i}\left({1}\right)={Y}_{j}\left({0}\right)\right)$, which quantifies the probability that treatment improves outcomes for a randomly selected pair of individuals, $\left(i,j\right)$. Zhang et al. [38] proposed outcome-regression estimators for $\theta$, but their approach relies on parametric modeling and often requires outcome transformations to achieve normality—an assumption at odds with the transformation-invariance of rank-based methods.

To address these limitations, we propose a semiparametric outcome-regression estimator based on the cumulative probability model (CPM). Although CPMs are commonly used for ordinal outcomes, Liu et al. [15] showed that they naturally extend to continuous and mixed-type outcomes by estimating an unspecified monotone transformation. This flexibility makes CPMs well suited for modeling skewed outcomes without requiring parametric distributional assumptions. Recent theoretical work [14] further established asymptotic properties of CPMs for continuous outcomes, motivating their use in causal estimation.

In this paper, we develop a CPM-based estimator of the Mann–Whitney-type causal effect and evaluate its performance through simulation studies and an application to kidney function outcomes in an observational cohort of adults living with HIV in Northern Nigeria.

## Methodology

### Mann–Whitney-type causal effect estimand

We consider a setting with two treatment groups: the exposed (A = 1) and the unexposed (A = 0). The outcome Y is assumed to be orderable, meaning it may be continuous, ordinal, or a mixture of both. We define ${Y}_{i}\left(1\right)$ as the potential outcome for individual *i* if assigned A = 1 and ${Y}_{i}\left(0\right)$ as the potential outcome for individual *i* if assigned A = 0. Additionally, let X be a vector of covariates that are unaffected by treatment. Our focus is on estimating a population level Mann–Whitney-type causal effect.

Let ${F}_{Y\left(0\right)}(y)$ and ${F}_{Y\left(1\right)}(y)$ be the marginal distributions of *Y*(0) and *Y*(1), respectively. The Mann–Whitney-type effect estimand is represented in terms of the marginal distributions of potential outcomes as:

1 $$\theta =E\left\{h\left({Y}_{i}\left(1\right),{Y}_{j}\left(0\right)\right)\right\}=\iint h\left({y}_{1},{y}_{0}\right)d{F}_{Y\left(1\right)}\left({y}_{1}\right)d{F}_{Y\left(0\right)}\left({y}_{0}\right)$$

where

2 $$h\left({y}_{1},{y}_{0}\right)=I\left({y}_{1}>{y}_{0}\right)+0.5I\left({y}_{1}={y}_{0}\right)$$

and $I\left(.\right)$ is an indicator function. For a continuous outcome, $h\left({y}_{1},{y}_{0}\right)=I\left({y}_{1}>{y}_{0}\right)$ and the corresponding θ, namely, $P\left({Y}_{i}\left(1\right)>{Y}_{j}\left(0\right)\right)$ captures the tendency of a randomly selected unit, subject *i*, to have a higher potential outcome under treatment than the potential outcome under control of an independent randomly selected unit, subject *j.* Note that θ depends only on marginal distributions of the potential outcomes because subscripts *i* and *j* represent two independent subjects.

Because $\theta$ depends on the marginal distributions of unobserved potential outcomes, identification requires expressing ${F}_{Y(a)}\left(y\right)\mathrm{=}P\left(Y\left(a\right)\le y\right)$ in terms of observable data. We rely on four standard causal assumptions:

1. *No interference*: One individual’s treatment does not affect another’s potential outcomes.
2. *Consistency*: The potential outcome for an individual under a specific treatment condition matches the observed outcome when that individual receives that treatment: $Y=Y(a)$ if $A=a$.
3. *Ignorability*: Conditional on covariates X, treatment assignment is independent of the potential outcomes [24]: $Y(a)\perp\!\!\!\perp A\vert X$. This is an assumption of no unmeasured confounding.
4. *Positivity*: For all covariate values in the support of X, both treatment groups occur with nonzero probability: $0<P\left(A=1|X=x\right)<1$.

Under these assumptions, the marginal distribution of each potential outcome is nonparametrically identified. Specifically,

3 $$\begin{aligned}{F}_{Y\left(a\right)}\left(y\right)&=\int P\left(Y\left(a\right)\le y|X=x\right)d{F}_{X}\left(x\right)\\&=\int P\left(Y\left(a\right)\le y|A=a,X=x\right)d{F}_{X}\left(x\right)\\&=\int P\left(Y\le y|A=a,X=x\right)d{F}_{X}\left(x\right),\end{aligned}$$

where the first equality is from the law of total probability (conditioning on X), the second equality is from *Ignorability*, and the third is from *Consistency*. Thus, both marginal potential-outcome distributions are expressible entirely in terms of observable quantities and are therefore identified. The causal estimand, *θ*, expressed by (1) is also identified.

The distribution of *X*, ${F}_{X}\mathrm{(}x\mathrm{)}$ can be estimated without modeling assumptions using the empirical distribution of *X*. However, with continuous or multi-dimensional *X*, modeling assumptions will typically be needed to estimate $P\left(Y\le y\mathrm{|}A\mathrm{=}a\text{, }X\mathrm{=}x\right)$. Ideally, to maintain the spirit of the rank-based nature of *θ*, the model for $P\left(Y\le y\mathrm{|}A\mathrm{=}a\text{, }X\mathrm{=}x\right)$ will also be based on ranks. In the next section, we describe such a model.

### Semiparametric cumulative probability model

To estimate the marginal distributions of the potential outcomes $Y\left(1\right)$ and $Y\left(0\right)$, we model the conditional distribution of the observed outcome $Y$ given covariates $X$ and treatment $A$ using a CPM. CPMs are flexible regression models for ordered or continuous outcomes that directly model the cumulative probability $P\left(Y\le y|A=a, X=x\right)$. The CPM is well-suited to our setting because it is *rank-based*, does not require specifying a parametric distribution for *Y*, and aligns naturally with the Mann–Whitney-type estimand, which depends only on the ordering of outcomes.

We assume that *Y* follows a semiparametric linear transformation model of the form

$$Y=H(\beta^TX+\mathrm\uptau A+\varepsilon\mathit),$$

where $H(.)$ is an unknown monotone increasing transformation of the outcome, $\beta$ is a vector of covariate coefficients, $\tau$ is the coefficient associated with the treatment variable, and ε is a random variable that is assumed to follow a cumulative distribution function ${F}_{\varepsilon }$ and is independent of *X* and *A*. For ease of presentation, we have chosen to write this model with simple linear relationships, but we note that the model could easily be made more flexible by including, for example, pre-specified functions of *X* (e.g., polynomials, splines, products) and interactions between functions of *X* and *A*. It follows that ${H}^{-1}\left(Y\right)={\beta }^{T}X+\tau A+\varepsilon$, where ${H}^{-1}\left(Y\right)$ is a transformation of *Y* such that the transformed value is linearly related to *X* and *A*. The cumulative probability distribution of *Y* conditional on *X* and *A* is

4 $$\begin{aligned}F\left(y|X,A\right)&=P\left(Y\le y|X,A\right)=P\left[H\left({\beta }^{T}X+\tau A+\varepsilon \right)\le y|X,A\right]\\&=P\left[\varepsilon \le {H}^{-1}\left(y\right){-\beta }^{T}X-\tau A|X,A\right]\\&={F}_{\varepsilon }\left({H}^{-1}\left(y\right){-\beta }^{T}X-\tau A\right)\end{aligned}$$

By letting ${H}^{-1}(y)=$ $\alpha \left(y\right)$ and *G*(.) = ${{F}_{\varepsilon }}^{-1}(.)$, we write the linear transformation model as a CPM:

5 $$G\left[P\left(Y\le y|X,A\right)\right]=\alpha \left(y\right){-\beta }^{T}X-\tau A,$$

where $\alpha \left(y\right)$ is sometimes referred to as an intercept function and *G*(.) is a link function (e.g., probit, logit, log–log, etc.). We fit our CPM in a semiparametric manner that uses a step function to estimate$\alpha \left(y\right)$. Without loss of generality, for *n* ordered unique observed values of *Y* denoted by *y*_i_, we estimate *n* associated intercepts, α_i_ = $\alpha \left({y}_{i}\right)$, i = 1, ⋯, *n*. The CPM thus estimates one intercept for each unique ordered outcome value. We express the CPM as:

6 $$G\left[P\left(Y\le y|X,A\right)\right]={\alpha }_{i}{-\beta }^{T}X-\tau A.$$

The nonparametric likelihood function of the CPM for independent and identically distributed realizations of $\left({X}_{i},{A}_{i},{Y}_{i}\right)$ is given by:

7 $$\begin{aligned}L\left(\beta ,\tau ,\alpha \right)&=\prod\nolimits_{i=1}^{n}\left\{{G}^{-1}\left({\alpha }_{i}{-\beta }^{T}{X}_{i}-\tau {A}_{i}\right)\right. \\& \left.-{G}^{-1}\left({\alpha }_{i-1}{-\beta }^{T}{X}_{i}-\tau {A}_{i}\right)\right\}\end{aligned}$$

where ${\alpha }_{0}$ is an auxiliary parameter with the constraint ${\alpha }_{0}$ <${\alpha }_{1}$. The likelihood function reaches its maximum when ${\alpha }_{0}$ = $-\infty$ and ${\alpha }_{n}$ =$\infty$. This likelihood is equivalent to the multinomial likelihood for cumulative link models when outcomes are considered as ordered categorical [18]. Maximizing the likelihood results in the nonparametric maximum likelihood estimators (NPMLEs) for$\alpha =({\alpha }_{1}, {\alpha }_{2}, ..., {\alpha }_{n-1}$),$\beta$, and τ. With a bounded range of *Y* and mild regularity conditions, the maximum likelihood estimators of $\alpha$,$\beta$, and $\tau$ are consistent and asymptotically normally distributed [14].

Because the transformation, ${H}^{-1}\left(Y\right)=\alpha \left(Y\right),$ is nonparametrically estimated, the estimated parameters τ and $\beta$ are invariant to any monotonic transformation of *Y*. Thus, the CPM is rank-based. The likelihood (7) is easily extended to incorporate ties [39]; CPMs can model continuous, ordinal, or mixtures of continuous and ordinal data (e.g., data with detection limits). With a single binary predictor *X*, the score test for the corresponding $\beta$ in the CPM is nearly identical to the Mann–Whitney/Wilcoxon rank sum test ([18, 39]). CPMs can be fit in a computationally efficient manner using the `orm` function in the `rms` package [8] in R software [22].

### Estimating Mann–Whitney-type causal effects using CPMs

Estimating the distributions of potential outcomes, ${F}_{Y(a)}(y)$, follows the maximum likelihood estimation of the model parameters. We do that by replacing the parameters in the RHS of (6) by their respective estimators. Then ${F}_{a}(y)$ can be estimated using a plug-in estimator for (3) as: 

for *a* = 0 or 1, *n* is the sample size, and $\widehat{\alpha }\left(y\right)$ is a step function with jumps at the observed $y$, specifically $\widehat{\alpha }\left(y\right)=\widehat{\alpha }\left({y}_{i}\right)={\widehat{\alpha }}_{i}$, where ${y}_{i}$=max{${y}_{1},\dots , {y}_{n}\}$ such that ${y}_{i}\le y$.

To estimate our Mann–Whitney causal effect parameter, θ, we use plug-in estimators for (1) based on the estimated marginal CDFs of the potential outcomes. Specifically,

9 $$\begin{array}{ll}{ \widehat{\theta }}_{REG}^{CPM}=\sum\nolimits_{i=1}^{n}\sum\nolimits_{j=1}^{n}h\left({y}_{i},{y}_{j}\right)\left[{\widehat{F}}_{1}({y}_{i})-{\widehat{F}}_{1}({y}_{i-1})\right] \left[{\widehat{F}}_{0}({y}_{j})-{\widehat{F}}_{0}({y}_{j-1})\right],\end{array}$$

where ${\widehat{F}}_{a}\left({y}_{0}\right)=0$ and ${\widehat{F}}_{a}\left({y}_{n}\right)=1$. An alternative formulation would exclude *i* = *j* from the double summation to avoid ties; with large *n*, this alternative is equivalent to Eq. (9), which we use throughout this manuscript.

Because the CPM parameter estimates are consistent and asymptotically normal [14], our CPM-based estimator is also consistent for θ and asymptotically normal. Specifically, under the causal assumptions (*no interference, consistency, ignorability,* and *positivity*), mild regularity assumptions, and a bounded range of *Y*,

$$\widehat\theta_{REG}^{CPM}\xrightarrow p\theta; \sqrt n\left(\widehat\theta_{REG}^{CPM}-\theta\right)\xrightarrow dN\left(0,\;\sigma_{\widehat\theta_{REG}^{CPM}}^2\right)$$

where ${\sigma }_{{\widehat{\theta }}_{REG}^{CPM}}^{2}$ is the asymptotic variance. We recommend estimating ${\sigma }_{{\widehat{\theta }}_{REG}^{CPM}}^{2}$ via the nonparametric bootstrap.

## Simulations

### Simulation procedure

We conducted a simulation study to evaluate the finite-sample performance of the proposed CPM-based estimator. For each scenario, we generated 1,000 datasets with sample sizes $n\in \left(\text{50, 200, 500}\right)$. Each dataset included a normally distributed covariate *X* $\sim \, N\mathrm{(0,1)}$, a binary treatment *A*, and a right-skewed continuous outcome *Y*. Treatment was assigned according to the logistic model

$$P\mathit(A=1\vert\boldsymbol X\mathit)=expit\mathit(\alpha_0+\alpha_1X\mathit),$$

with ${\alpha }_{1}=0.75$ chosen to induce moderate confounding while maintaining adequate overlap, and ${\alpha }_{0}=-0.5$ selected to center the marginal treatment probability near 0.4. Outcomes were generated from

$$Y\mathrm{=exp(}\beta X\mathrm{+}\tau A\mathrm{+}\varepsilon \mathrm{),}$$

where $\varepsilon \sim \, N\mathrm{(0,1).}$ The exponential transformation induces right-skewness, and the parameters $\beta \in \left(\text{0, 0.5, 2}\right)$ and $\tau \in \left(\text{0, 0.5, 2}\right)$ control confounding strength and treatment effect magnitude, respectively. These combinations produce a wide range of true Mann–Whitney parameters. When $\tau = \mathrm{0}$, the true causal effect, $\theta$, equals 0.5; for $\tau \ne {0}$, the true value of $\theta$ was computed using large Monte Carlo samples (Supplementary Material).

#### Estimation

The causal Mann–Whitney parameter was estimated using the statistical methods described in "Methodology" section. CPMs were fit using a probit link function (the inverse standard normal CDF), while leaving the transformation function *H(.)* unspecified and estimating it nonparametrically via the orm() function in the rms package [8] in R [22]. Ninety-five percent confidence intervals were constructed using 200 bootstrap replicates, with the interval defined as $\widehat{\theta }\pm \mathrm{1.96}\times {\widehat{SD}}_{\mathrm{bootstrap}}$.

To assess robustness, we also fit CPMs under two forms of misspecification: 1) *Incorrect link function* (logit instead of probit) and 2) *Omission of the confounder X* (i.e., constraining its coefficient to zero). We compared the CPM estimator with two parametric outcome-regression estimators following Zhang et al. [38]: 1) *Correctly specified model*: log-transforming *Y* before fitting a normal linear model. 2) *Misspecified model*: applying an inappropriate square-root transformation and then fitting a normal linear model. These comparators represent idealized and realistic misspecification settings, respectively. Full details appear in Supplementary Material.

#### Performance metrics

For each scenario, we computed bias (the difference between the average of our estimated values and true values), the standard deviation (SD) of estimates across the 1000 simulated datasets, the root mean squared error (RMSE), and the coverage probability (CP) of 95% confidence intervals.

#### Software and computational environment

All analyses were conducted using R Software Version 4.4.1 [22] and interface RStudio Version 4.2.7 [25] on a Windows 11 Pro platform [19].

### Simulation results

Simulation results are presented in the figures that follow. The confounder and treatment coefficients in the models are represented by *β* and *τ*, respectively.

#### Performance metrics of estimators from correctly specified CPMs

**Fig. 1:**
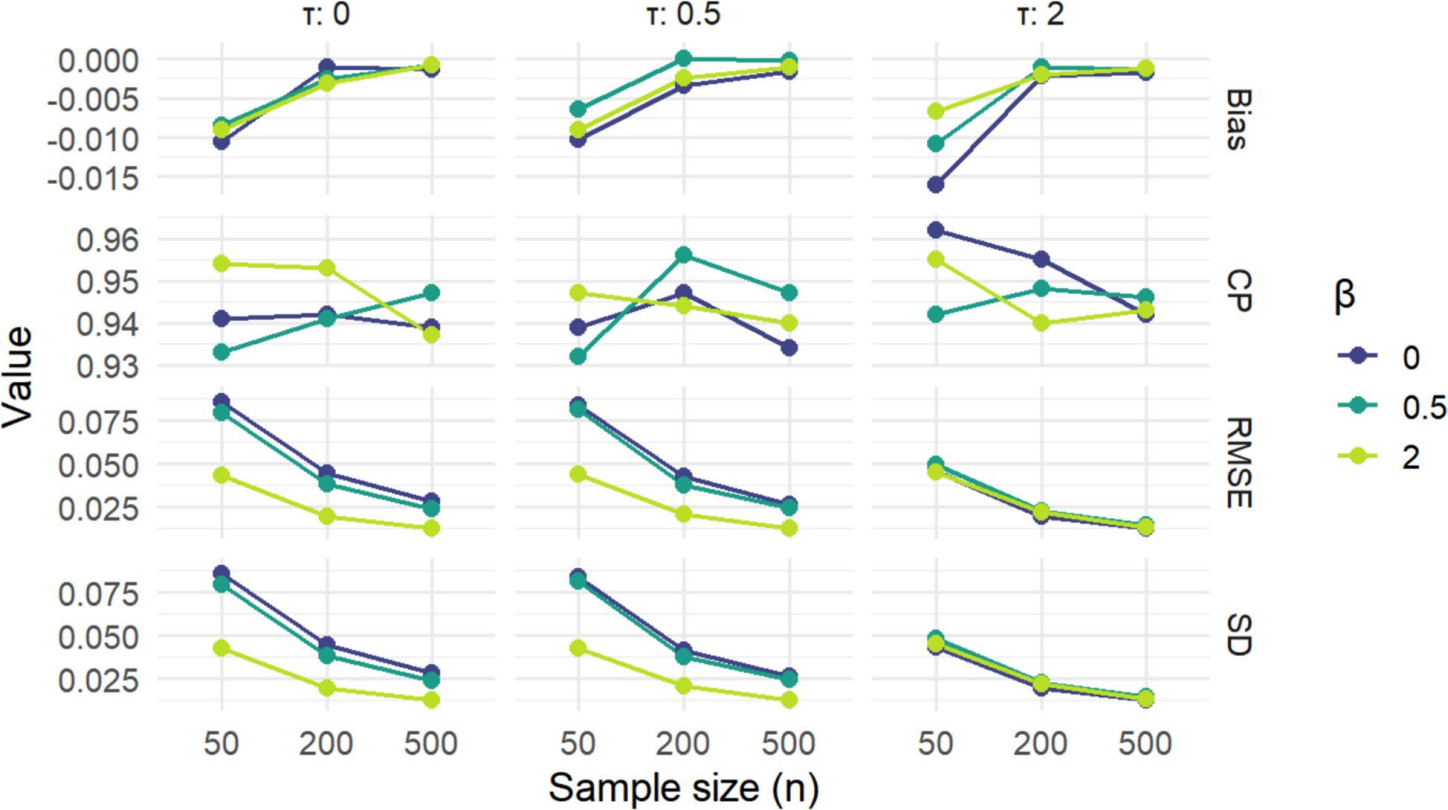
Correctly specified CPMs

Figure 1 summarizes the performance of our method with properly specified CPMs across multiple sample sizes, levels of confounding, and treatment effect sizes. Across all panels, increasing the sample size steadily improves estimator behavior: bias shrinks toward zero, coverage probabilities move closer to the nominal 95% level, and both RMSE and SD decline as sample size increases. Larger values of β and τ consistently make estimation more challenging, producing slightly more bias and variability in smaller samples, but these differences narrow as *n* grows. By *n* = 500, the curves for all parameter settings converge, indicating that the estimator is consistent and stabilizes well in large samples.

#### Performance metrics of estimators from link function misspecified CPMs

**Fig. 2:**
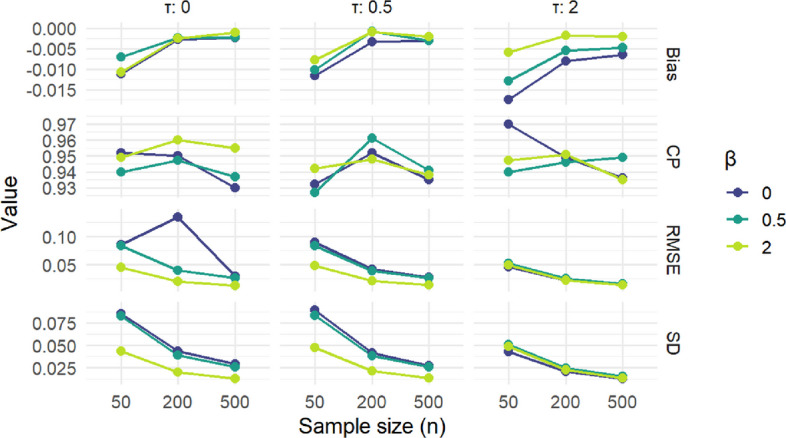
Link function missecified CPMs

Figure 2 presents the performance of our method using the improperly specified logit link function, rather than probit, for the cumulative probability models. Bias remained low, coverage probability was still close to the 0.95 mark, at all sample sizes, with small deviations depending on *β* and *n*. SD and RMSE decreased with increasing *n*. Overall, these results demonstrate the estimator’s robustness to misspecification of the link function.

#### Performance metrics of estimators from linear predictor misspecified CPMs

**Fig. 3:**
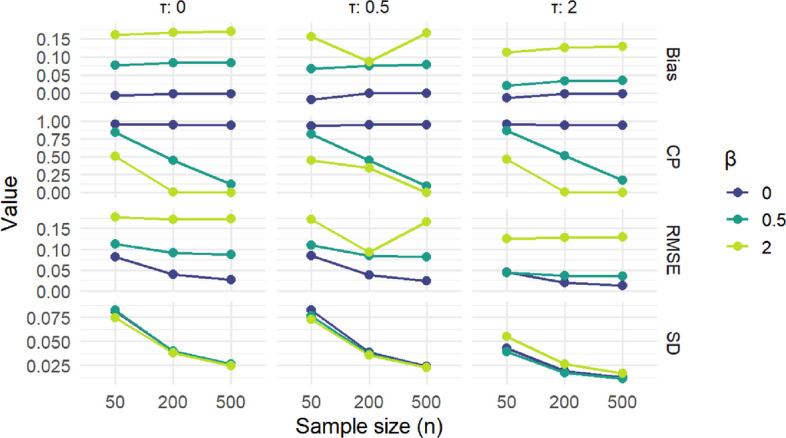
Linear predictor misspecified CPMs

Figure 3 shows the performance of our method when employing improperly specified linear predictors, neglecting to include the confounder variable, for the cumulative probability models. As expected, bias was high and coverage low except in the scenario where the confounder effects were absent (i.e., *β* = 0). These findings underscore the necessity of properly accounting for confounders to maintain estimator validity.

#### Bias and relative efficiency of the CPM-based estimator vs transformed parametric model-based estimators

**Fig. 4:**
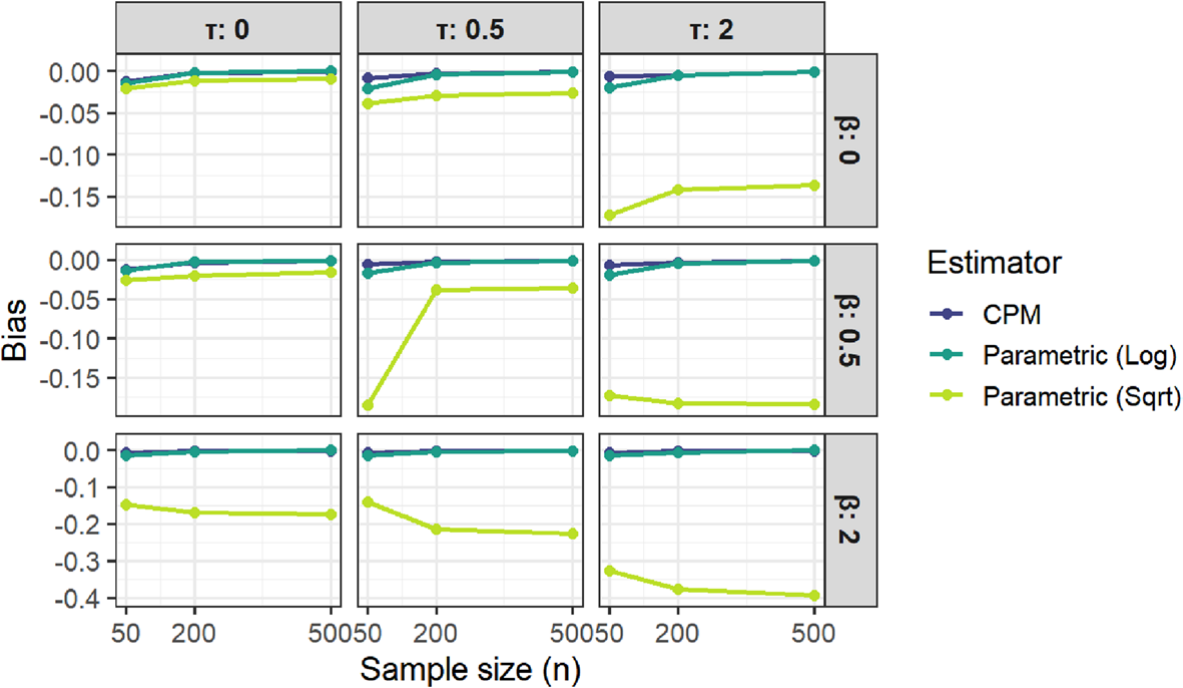
Bias Comparison (CPM vs Parametric Estimators)

Figure 4 illustrates how the bias of the CPM-based estimator compares with that of parametric estimators across all combinations of $\beta$, $\tau$, and *n*. The CPM estimator shows consistently small bias in every setting, closely matching the performance of the correctly transformed parametric model when the transformation is appropriate. In contrast, the square-root–based estimator exhibits substantial negative bias in many scenarios, particularly when either $\beta$ or $\tau$ is large, reflecting the consequences of misspecification. Bias decreases with increasing sample size for all estimators, but the CPM and correctly specified parametric estimator converge much more rapidly, whereas the misspecified estimator remains biased even at larger $n$.

**Fig. 5:**
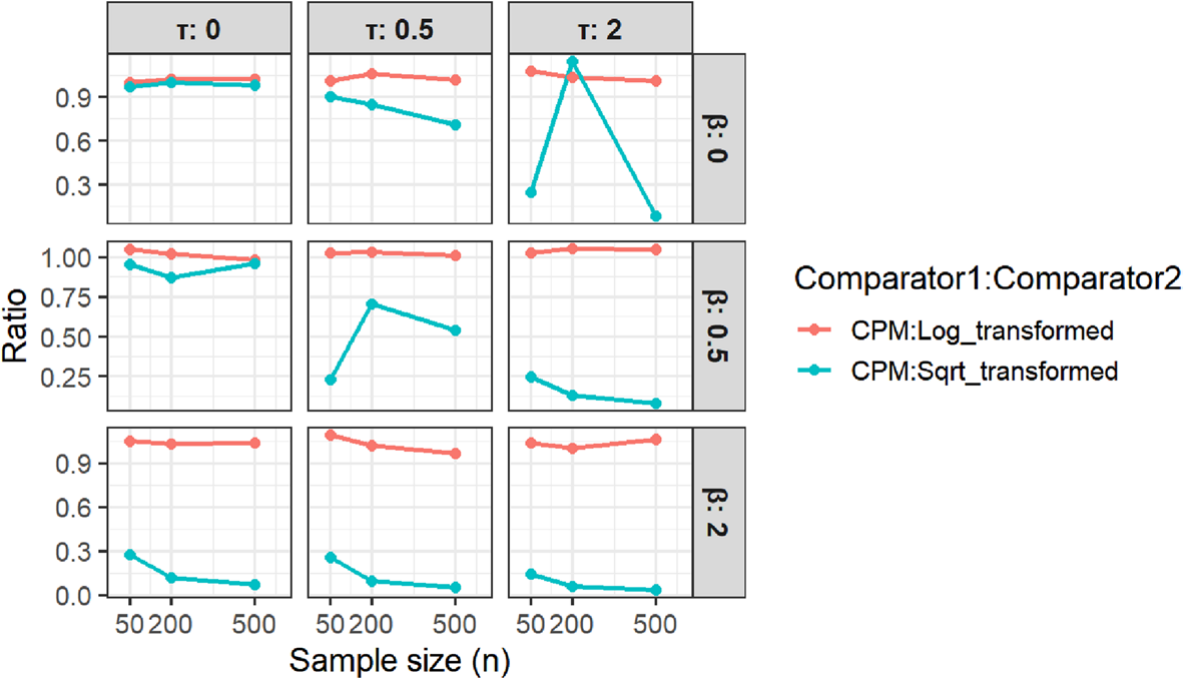
Efficiency Comparison (RMSE Ratios)

Figure 5 displays the ratio of RMSEs for CPM-based estimators relative to parametric estimators across the same grid of $\beta$, $\tau$, and *n*. When the parametric model is correctly specified (log transformation), RMSE ratios tend to be close to 1, indicating similar efficiency, with CPM estimators generally slightly less efficient. However, when the parametric model is misspecified (square-root transformation), RMSE ratios fall well below 1 across most panels. The improvement of CPM-based estimators over misspecified parametric estimators is most pronounced when $\beta$ or $\tau$ is large.

Taken together, the Figs. 4 and 5 highlight the robustness of the CPM approach: it achieves low bias comparable to a correctly specified parametric model without much loss in efficiency while avoiding the severe bias that arises when the parametric transformation is incorrect.

## Application

We applied the proposed method to data from the *Etiology of Persistent Microalbuminuria in Nigeria (P-MICRO) Study*, an observational cohort designed to estimate the causal effect of HIV on urine albumin-to-creatinine ratio (uACR) and estimated glomerular filtration rate (eGFR) among adults receiving care at Aminu Kano Teaching Hospital (AKTH) in Northern Nigeria.

The P-MICRO study was initiated following findings from the *Renal Risk Reduction (R3) Trial*, which reported high rates of persistent albuminuria among ART-treated people with HIV (PWH) [35]. Microalbuminuria is a well-established risk factor for cardiovascular and kidney disease and a marker of early end-organ damage in both the general population and PWH [6, 11, 16, 17]. Defined as a uACR of 30–300 mg/g, microalbuminuria reflects early glomerular injury or microvascular dysfunction [1, 23]. HIV infection may increase the risk of albuminuria through HIV-associated nephropathy, chronic inflammation, comorbidities, and potential nephrotoxic effects of antiretroviral therapy. Prior studies have linked albuminuria in PWH to heightened immune activation, faster progression to AIDS, and increased mortality [2, 7, 10, 21, 28, 36].

The P-MICRO cohort includes ART-treated PWH and age- and sex-matched HIV-negative adults recruited from AKTH [34]. Our analysis uses baseline data from 2,998 participants, of whom 2,248 (75%) are PWH. The exposure is HIV status (positive vs. negative), and the outcomes are uACR and eGFR—standard markers of albuminuria and kidney function. As expected, uACR values were right-skewed [12, 32], making a Mann–Whitney-type estimand appropriate (Fig. 1, Supplementary Material). eGFR exhibited left-skewness, consistent with population-level patterns ([3, 30]; Fig. 2, Supplementary Material). Although categorizing uACR is generally discouraged, we also analyzed the ordered categories (normo-, micro-, and macroalbuminuria corresponding to uACR <30, 30-300, and >300, respectively) to illustrate the flexibility of the CPM.

We adjusted for a rich set of baseline covariates selected to satisfy conditional exchangeability: age, BMI, ethnicity, sex, alcohol use, smoking status, hepatitis B infection, APOL1 risk genotype, hypertension category, diabetes, concomitant medication use, and other comorbidities. These variables reflect known demographic, clinical, and genetic determinants of kidney disease in PWH [28, 37].

To interpret the estimated Mann–Whitney parameters causally, we rely on standard assumptions for observational studies. First, we assume *no interference*, meaning that one participant’s HIV status does not affect another participant’s kidney outcomes—a reasonable assumption given the individual-level nature of HIV infection and kidney function. Second, we assume *ignorability*, i.e., that there are no unmeasured confounders between HIV status and kidney outcomes after adjusting for the rich set of demographic, behavioral, clinical, and genetic covariates described above. These variables were selected based on prior evidence identifying demographic, clinical, and genetic determinants of kidney disease among people living with HIV [28, 37]. Nonetheless, as with any observational study, residual unmeasured confounding cannot be ruled out, and causal interpretations should be viewed in that context. Finally, we assume *positivity*, meaning that individuals with each covariate pattern have a nonzero probability of being HIV-positive or HIV-negative; this assumption is supported by the recruitment of matched HIV-negative adults in the P-MICRO study, and no evidence of covariate separation across HIV status. Together, these assumptions define the conditions under which our CPM-based estimates can be interpreted as causal effects of HIV on uACR and eGFR.

Tables 1, 2 and 3 compare the performance of Mann–Whitney-type effect estimators using different modelling approaches (CPMs with various link functions and parametric models with different transformations) to evaluate the effect of HIV on uACR and eGFR adjusted for potential confounders. To balance computational feasibility with empirical stability, we used 50 bootstrap replications to compute 95% confidence intervals (CI); increasing to 100 bootstrap replications for a subset of models produced nearly identical standard errors.

**Table 1:** Comparison of Mann–Whitney-type Effect Estimates from Different CPMs and Parametric Models for Assessing the Impact of HIV on uACR as a Continuous Variable

Model	Link/Transform	Estimate	SE	95% CI
CPM-logit	Logit	0.544	0.014	(0.516, 0.572)
CPM-probit	Probit	0.544	0.014	(0.516, 0.571)
CPM-loglog	Log–log	0.588	0.012	(0.564, 0.612)
NLM	None	0.178	0.004	(0.170, 0.186)
NLM-log	Log	0.574	0.012	(0.551, 0.596)
NLM-sqrt	Sqrt	0.344	0.010	(0.324, 0.365)

*CPM* cumulative probability model, *NLM* normal linear model assumed after transformation

**Table 2:** Comparison of Mann–Whitney-type effect estimates from different CPMs and parametric models for assessing the impact of HIV on uACR as a categorical variable

Model	Link/Transform	Estimate	SE	95% CI
CPM-logit	Logit	0.544	0.007	(0.530, 0.557)
CPM-probit	Probit	0.545	0.007	(0.533, 0.558)
CPM-loglog	Log–log	0.547	0.006	(0.535, 0.560)

*CPM* cumulative probability model, *NLM* normal linear model assumed after transformation

**Table 3:** Comparison of Mann–Whitney-type effect estimates from different CPMs and parametric models for assessing the impact of HIV on eGFR

Model	Link/Transform	Estimate	SE	95% CI
CPM-logit	Logit	0.340	0.013	(0.315, 0.366)
CPM-probit	Probit	0.346	0.013	(0.320, 0.372)
CPM-loglog	Log–log	0.408	0.011	(0.386, 0.430)
NLM	None	0.343	0.009	(0.325, 0.361)
NLM-log	Log	0.358	0.010	(0.338, 0.378)
NLM-sqrt	Sqrt	0.347	0.013	(0.322, 0.372)

*CPM* cumulative probability model, *NLM* normal linear model assumed after transformation

With uACR as a continuous outcome variable, CPM-based estimators provided very similar effect estimates across different link functions, with logit and probit models yielding nearly identical results. The log–log link function resulted in the best model fit (e.g., highest likelihood; see Supplementary Material). In contrast, parametric models showed variability, highlighting their sensitivity to transformation choice. Among them, NLM-log produced estimates closest to CPMs, suggesting a log transformation may have been most appropriate. Overall, CPMs offer a robust and stable analysis approach, while parametric models require careful transformation selection to ensure reliable interpretations.

When uACR was categorized, the Mann–Whitney-type effect estimates remained very similar across link functions, closely resembling their continuous counterparts. This stability validates the reliability of CPM-based estimators in estimating causal effects, regardless of whether the outcome variable is treated as continuous or categorical. This finding supports the idea that CPMs provide a flexible framework for evaluating the effect of HIV on kidney function markers, without requiring parametric assumptions.

Our Mann–Whitney causal effect estimators based on the CPM suggest that HIV lowers eGFR (lower eGFR implies reduced kidney function.) This conclusion held, regardless of the link function used for the CPM. The estimated Mann–Whitney causal effect was slightly higher using the log–log link function. It should be noted that the logit link function resulted in the best model fit (e.g., highest likelihood; see Supplementary material). Interestingly, parametric models produced similar Mann–Whitney causal effect estimates to CPM-based estimators in this case. This suggests that the relationship between HIV and eGFR may be less dependent on transformation choices compared to uACR. The agreement between approaches reinforces the validity of the observed negative effect of HIV on kidney function.

## Discussion

In this study, we proposed a semiparametric outcome-regression estimator for the Mann–Whitney–type causal effect based on the CPM. Through simulations and an application to the P-MICRO study, we showed that the CPM-based estimator is robust, reliable, and well-suited for causal inference with skewed or non-normal outcomes.

Zhang et al. [38] proposed Mann–Whitney treatment effect estimators, providing researchers with a structured approach for estimating these causal effects. The outcome regression estimator of Zhang et al. [38] requires specifying a transformation and then assuming normality. These parametric assumptions can be restrictive and sensitive to misspecification. In contrast, our CPM-based outcome regression estimator is rank-based and consistent with the spirit of the Mann–Whitney test, which does not require specific distributional forms or transformations, thereby providing robustness. This robustness is desirable for practical applications where the true distribution of the data is generally unknown and often difficult to model accurately, particularly with skewed data.

Across simulation scenarios, the CPM estimator exhibited low bias, decreasing variance with larger samples, and coverage probabilities close to the nominal level when paired with bootstrap inference. The CPM estimator maintained low bias and high accuracy, even with strong confounders and treatment effects. Notably, the CPM-based estimators resulted in minimal loss of efficiency when compared to properly specified parametric models, suggesting there is little cost to using these semiparametric models instead of parametric models. Convergence failures were rare and occurred only in extreme simulation scenarios with strong confounding, large treatment effects, and link-function misspecification. In such settings, the working link function provides a poor approximation to the true conditional distribution, which can lead to flat or unstable likelihood surfaces and numerical non-convergence. In practice, non-convergence may therefore serve as a diagnostic for model misspecification. When it occurs, analysts can consider alternative link functions and revising the functional form of the linear predictor.

The CPM estimator demonstrated robustness to the misspecification of the link function; however, improper linear predictor specification resulted in poor performance. As with all causal models, care must be taken in selecting covariates and how to include them in regression models. CPMs offer a degree of flexibility over normal linear models because they do not require specifying a transformation of the outcome. However, like normal linear models, how the covariates are included in the models is important. In our simulations, we focused on a single confounder to isolate the estimator’s behavior, but in practice, multiple covariates—and potentially nonlinear or interacting effects—may influence the outcome. CPMs can incorporate flexible covariate structures, including splines, polynomial terms such as X^2^, and interactions, but misspecification of these components can introduce bias. Future work could evaluate the estimator’s performance under more complex covariate structures and explore strategies for data-adaptive specification of the linear predictor. Additionally, performance may be affected by limited sample sizes. Future research should assess performance under broader data-generating mechanisms and outcomes, and investigate computational refinements to improve scalability.

In settings with complex confounding structures—such as nonlinear covariate effects, interactions, or high-dimensional adjustment sets— doubly robust estimators are often preferable, where results are consistent if either the outcome regression model or a propensity score model is properly specified. While we did not consider doubly robust estimators in this manuscript, our outcome regression estimator based on CPMs could be incorporated into a doubly robust estimator such as that presented by Zhang et al. [38]. Such an extension would further enhance robustness in complex observational settings.

Our CPM-based approach differs in important ways from more commonly used propensity score methods such as propensity score matching (PSM) and inverse probability weighting (IPW). PSM and IPW estimate causal effects by modeling the treatment assignment mechanism and typically target mean-based estimands. These approaches require additional design choices—such as caliper selection, matching ratios, or weight stabilization—and can be sensitive to extreme propensity scores. In contrast, our CPM-based outcome regression estimator directly models the conditional distribution of the outcome and naturally targets Mann–Whitney–type estimands, which are particularly well suited for skewed outcomes such as uACR and eGFR. Because the CPM does not rely on propensity score modeling, it avoids challenges related to poor covariate overlap or extreme weights.

The application of the CPM estimator to the P-MICRO study data provided insight into the causal relationship between HIV status and albuminuria and kidney function among ART-treated PWH in Northern Nigeria. CPMs produced stable and consistent estimates for uACR across modeling choices and avoided the sensitivity to transformation assumptions observed with parametric models. The agreement between CPM-derived estimates for continuous and categorized uACR further supports their reliability. We found that ART-treated PWH had higher uACR, supporting the hypothesis that HIV increases the risk of albuminuria in PWH [20, 29], and lower eGFR than HIV-negative adults, consistent with prior evidence and underscoring the importance of early renal surveillance in HIV-positive populations.

Several limitations warrant consideration. Causal interpretation relies on standard identification assumptions—consistency, no interference, ignorability, and positivity. Although we adjusted for a rich set of demographic, behavioral, clinical, and genetic covariates, residual unmeasured confounding cannot be ruled out in the HIV application. Factors such as socioeconomic status, environmental exposures, health-seeking behavior, or unmeasured comorbidities may influence both HIV status and kidney outcomes.

In conclusion, this study contributes to the growing body of literature on causal inference methods by introducing a robust and practical tool for researchers investigating causal relationships in observational data.

## Supplementary Information

Supplementary Material 1.

## Data Availability

Simulation and data analysis code are publicly available at https://github.com/shepheb1/ArchivedAnalyses. Because of privacy concerns and data sharing laws/agreements, we cannot freely share the dataset used for the kidney disease study; these data can be made available upon request by contacting the study authors and completing necessary data use agreements. We adhere to the journal’s ethical and archiving policies.
