# Supplementary material for: Semiparametric outcome regression-based estimator of Mann–Whitney-type causal effect
Source: BMC Med Res Methodol. 2026 Mar 26;26:104. doi: 10.1186/s12874-026-02840-1 (PMC13141289; doi:10.1186/s12874-026-02840-1)
Supplement: Supplementary file 1 — Supplementary Material 1. [file 12874_2026_2840_MOESM1_ESM.pdf]

Supplementary Materials for "Semiparametric Outcome Regression-Based Estimator of the Mann-Whitney-type Causal Effect"

Web Appendix I: Simulation Approach for Estimation of True Theta (θ)

A pseudo-population of n=1,000,000 observations was generated according to the study's data-generating process to estimate true values across various simulation scenarios. The methodology involved two steps:

Step 1: Parameter Specification and Data Generation

- n set to 1,000,000.
- Confounder coefficient (β) and treatment effect parameter (τ) selected
- Confounder sampled from a standard normal distribution
- Potential outcomes under both treatment and control generated using the exponential linear model with added noise as described in Section 3.1.

Step 2: Estimate Causal Effects via Pairwise Comparison

- Randomly sampled 1,000,000 independent pairs of potential outcomes (i, j)
- Calculated $h(y_i, y_j)$ across all sampled pairs; $h(y_i, y_j) = 1$ if individual i had a greater outcome than j, $h(y_i, y_j) = 0.5$ if their outcomes were equal, and $h(y_i, y_j) = 0$ if individual i had a smaller outcome than j.
- Averaged results across all pairs to estimate the population-level Mann–Whitney-type causal effect

Web Appendix II: Comparison Models

1. CPMs with correct link function:

   $G[P(Y \leq y|X,A)]=\alpha(y)-\beta_1{}^T X-\beta_2 A$

   where G[.] is a probit link function
2. CPMs with incorrectly specified link function:

   $G[P(Y \leq y|X,A)]=\alpha(y)-\beta_1{}^T X-\beta_2 A$

   where G[.] is a logit link function
3. CPMs that exclude the confounder variable:

   $G[P(Y \leq y|X,A)]=\alpha(y)-\beta_2 A$

   where G[.] is a probit link function
4. Correctly transformed parametric models:

   $\log(Y)= \beta_0 +\beta_1 X + \beta_2 A + \varepsilon$, $\varepsilon \sim N(0, \sigma^2)$
5. Incorrectly transformed parametric models:

   $sqrt(Y)=\beta_0 +\beta_1 X + \beta_2 A + \varepsilon,$ $\varepsilon \sim N(0, \sigma^2)$

Note that the intercept, $\alpha(y)$, is estimated using a step function with the CPM. β_1 is the coefficient for the confounder variable X, β_2 is the coefficient for the treatment variable A, and ε is the error term, which accounts for the randomness or other factors not included in the model.

Web Appendix III: Figures

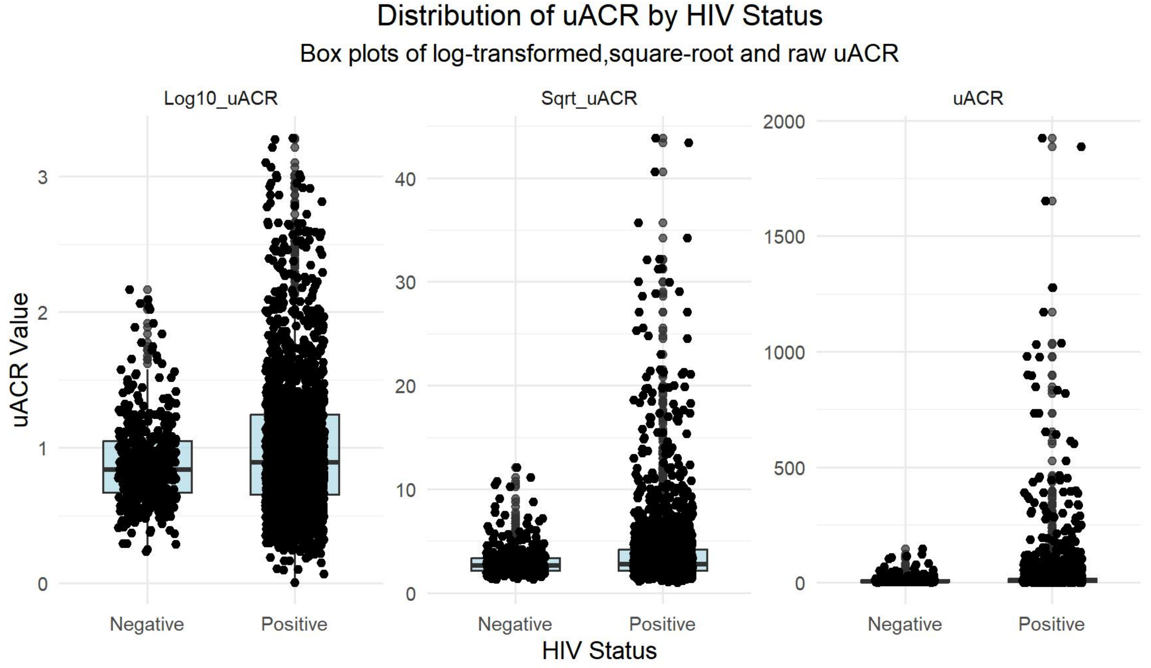

Figure 1: Boxplot of uACR across HIV status groups

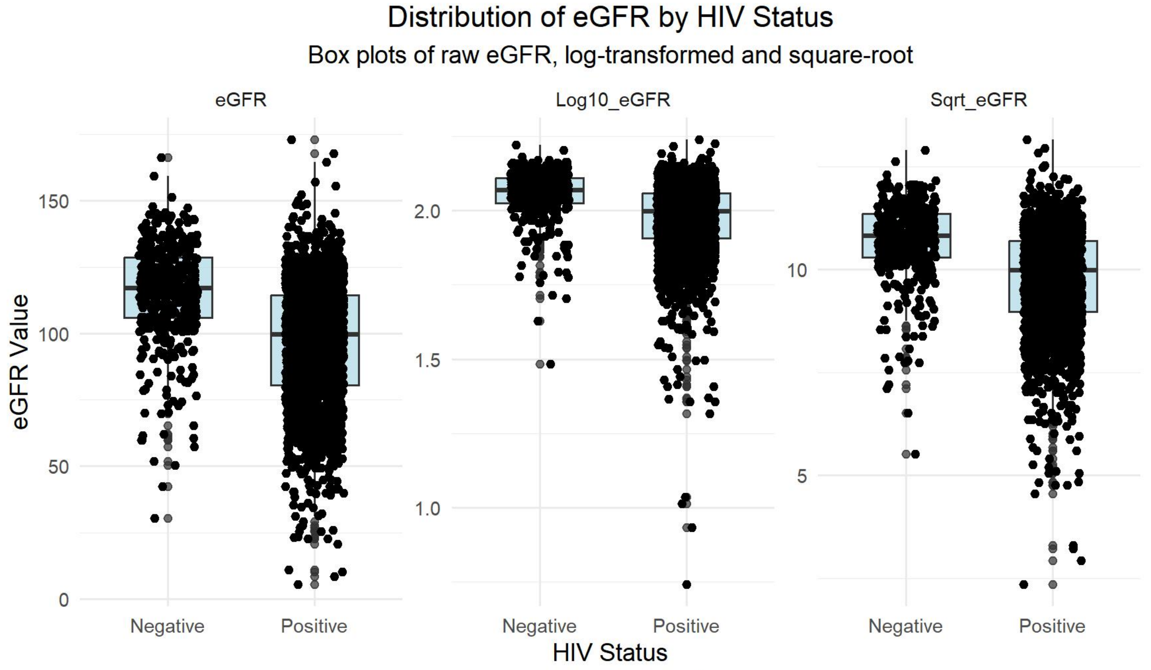

Figure 2: Boxplot of eGFR across HIV status groups

Web Appendix IV: R Codes

Function to fit cumulative probability model, predict F_Y and compute $\hat{\theta}$

```
library(rms)
compute_theta <- function(model_family, formula, data) {
  data_dd <- datadist(data); options(datadist = "data_dd")
  fit <- tryCatch(
    orm(formula, family = model_family, x=TRUE, y=TRUE, data = data),
    error = function(e) {
      message("Error fitting model: ", conditionMessage(e))
      return(NULL)
    }
  )
  if (is.null(fit)) return(NULL)
  # Auxiliary function to compute F(y|X)
    get_cdf <- function(fit_obj, newdata, ygrid) {
    lp <- predict(fit_obj, newdata=newdata, se.fit=TRUE)
    d <- ExProb(fit_obj)
    fy <- d(lp$linear.predictors, newdata, y=ygrid, conf.int=0.95)
    F_cdf <- 1 - colMeans(fy$prob)
    return(F_cdf)
  }
  newdat <- data.frame(X = data$X, A = 1) # Prepare new data with A=1 and A=0
  newdat0 <- data.frame(X = data$X, A = 0)
  ygrid <- sort(unique(data$Y))
  F1 <- get_cdf(fit, newdat, ygrid)
  F0 <- get_cdf(fit, newdat0, ygrid)
  p1 <- diff(c(0, F1))
  p0 <- diff(c(0, F0))
    # Compute Theta estimate
  h <- function(y1, y0) (y1 > y0) + 0.5 * (y1 == y0)
  temp <- matrix(NA,nrow = length(ygrid), ncol = length(ygrid) )
  for (j in 1:length(ygrid)){
    for(k in 1:length(ygrid)){
      temp[j,k] <- h(ygrid[j],ygrid[k])*p1[j]*p0[k]
    }
  }  Theta <- sum(temp)
      return(Theta)
  }
```

Function to fit transformed linear model, predict, and calculate $\hat{\theta}$

```r
compute_theta_lm <- function(data, formula, A, X, Y, n = NULL,
                             by_interval = 0.01, transform_Y = identity) {
  data$Y <- as.numeric(as.character(data$Y)) # Ensure Y is numeric
  data$Y_transformed <- transform_Y(data$Y) # Apply transformation

  # Fit using the transformed outcome
  formula <- as.formula(paste("Y_transformed ~", paste(c(A, X),
                                                        collapse = " + ")))
  fit <- lm(formula, data = data)  # Fit the linear model with transformed Y
  if (is.null(n)) { # Determine 'n' if not provided
    n <- nrow(data)
  }
  # Sequence of ys for integration
  ys <- seq(from = min(data$Y_transformed) - 0.5,
            to = max(data$Y_transformed) + 0.5, by = by_interval)
  sigma <- sd(fit$residuals)
  # Prepare matrices to store density values
  p1 <- matrix(NA, nrow = n, ncol = length(ys))
  p0 <- matrix(NA, nrow = n, ncol = length(ys))
  # For each observation, predict at A=1 and A=0
  for (i in 1:n) {
    # Create new data with A=1 and A=0 for the i-th observation
    new1 <- data[i, c(X, A), drop=FALSE]; new1[[A]] <- 1
    new0 <- data[i, c(X, A), drop=FALSE]; new0[[A]] <- 0
    mu1 <- predict(fit, newdata = new1) # Predict mean with model
    mu0 <- predict(fit, newdata = new0)
    p1[i, ] <- dnorm(ys, mean = mu1, sd = sigma) # Calculate density at each ys
    p0[i, ] <- dnorm(ys, mean = mu0, sd = sigma)
  }
  pdf1 <- colMeans(p1) # Average densities
  pdf0 <- colMeans(p0)
  # Construct H matrix for integration
  h.matrix <- matrix(0, nrow=length(ys), ncol=length(ys))
  diag(h.matrix) <- 0.5
  h.matrix[lower.tri(h.matrix)] <- 1  # rectangle rule approximation
  # Compute Theta
  Theta <- sum(pdf1 %*% h.matrix %*% pdf0) * (by_interval^2)
  return(Theta)
}
```

Web Appendix V: Model Fit and Residual Diagnostics

1. Model Fit Comparison Using Log-Likelihood

We compared loglikelihoods of cumulative probability models (CPMs) with different link functions for modeling uACR and eGFR:

**Model**	**Details**	**uACR Log-likelihood**	**eGFR Log-likelihood**
CPM 1	Logit link function	-17780.88	-16044.71
CPM 2	Probit link function	-17777.09	-16064.85
CPM 3	Log-log link function	-17765.36	-16135.79

With respect to uACR, the Log-log link yielded the highest log-likelihood, indicating the best statistical fit among the cumulative probability models—though the margin of improvement was modest. For eGFR, the Logit link clearly outperformed the alternatives, while the Log-log link showed the poorest fit.

2. QQ Plots of Empirical Residuals

We generated QQ plots of empirical residuals against a Uniform distribution for each model to detect departures from the assumption of no systematic error across the entire support. For the uACR models, all three link functions resulted in similar QQ plots. For the eGFR models, the logit link function most closely approximated the uniform distribution; minimal departures from uniformity are seen with the log-log link function.

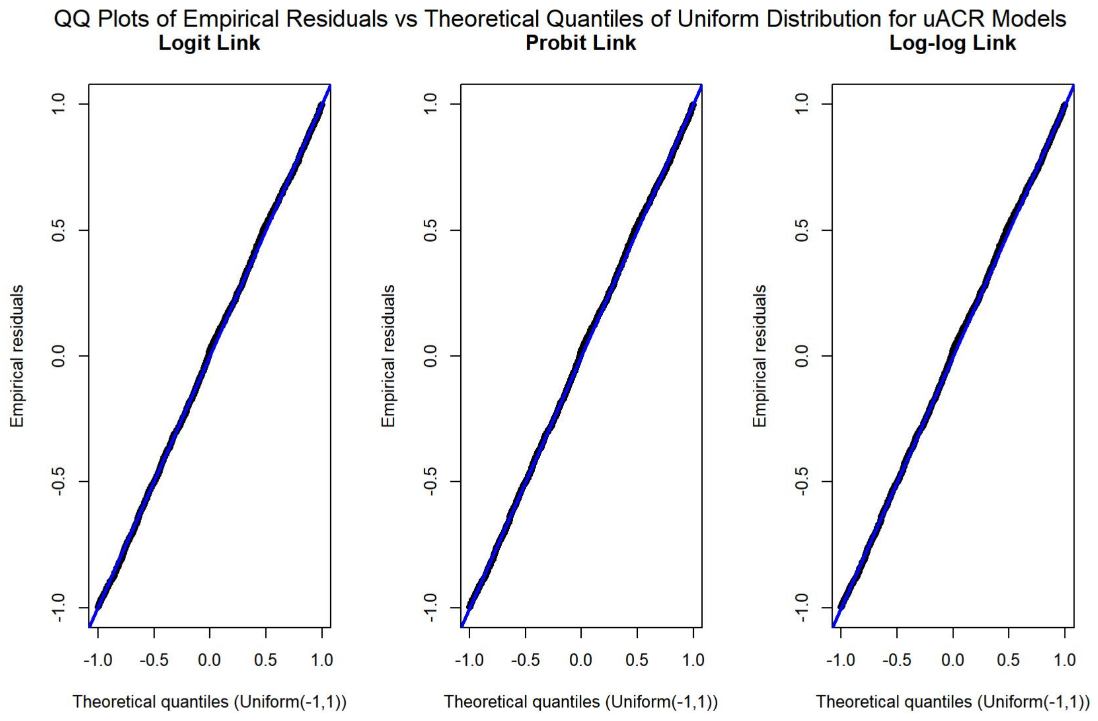

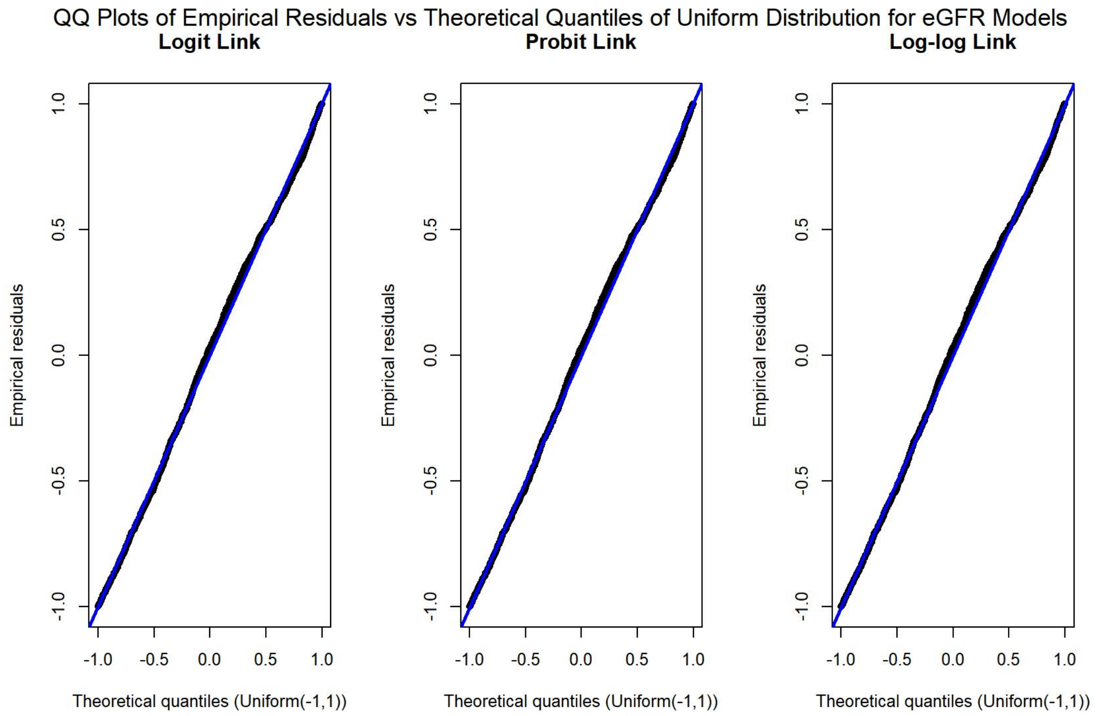

Additional Notes

- Bootstrapped estimates of treatment effects were also generated and are available upon request.
